# Metabolically Healthy versus Unhealthy Morbidly Obese: Chronic Inflammation, Nitro-Oxidative Stress, and Insulin Resistance

**DOI:** 10.3390/nu10091199

**Published:** 2018-09-01

**Authors:** Adriana Florinela Cӑtoi, Alina Elena Pârvu, Andra Diana Andreicuț, Aurel Mironiuc, Alexandra Crӑciun, Cornel Cӑtoi, Ioana Delia Pop

**Affiliations:** 1 Department of Pathophysiology, Faculty of Medicine, ‘Iuliu Hațieganu’, University of Medicine and Pharmacy, Cluj-Napoca 400012, Romania; parvualinaelena@umfcluj.ro (A.E.P.); cecan.andra@umfcluj.ro (A.D.A.); 2 Second Surgical Clinic, Faculty of Medicine, ‘Iuliu Hațieganu’, University of Medicine and Pharmacy Cluj-Napoca 400012, Romania; aurel.mironiuc@umfcluj.ro; 3 Department of Biochemistry, Faculty of Medicine, ‘Iuliu Hațieganu’, University of Medicine and Pharmacy, Cluj-Napoca 400012, Romania; acraciun@umfcluj.ro; 4 Department of Pathology, Faculty of Veterinary Medicine, University of Agricultural Sciences and Veterinary Medicine, Cluj-Napoca 400012, Romania; cornel.catoi@usamvcluj.ro; 5 Department of Exact Sciences, Faculty of Horticulture, University of Agricultural Sciences and Veterinary Medicine, Cluj-Napoca 400012, Romania; popioana@usamvcluj.ro

**Keywords:** metabolically healthy morbidly obese, metabolically unhealthy morbidly obese, metabolic syndrome, chronic inflammation, nitro-oxidative stress, insulin-resistance

## Abstract

Metabolically heathy obesity is characterised by the presence of obesity in the absence of metabolic disturbances. The aim of our study was to analyse pro-inflammatory, nitro-oxidative stress, and insulin-resistance (IR) markers in metabolically healthy morbidly obese (MHMO) with respect to metabolically unhealthy morbidly obese (MUHMO) with metabolic syndrome (MS) and to identify the potential predictors of MS in the MHMO group. Two groups of MHMO and MUHMO with MS were analysed. We evaluated serum high sensitivity C reactive protein (hsCRP), tumor necrosis factor alpha (TNF-α), chemerin, nitrite and nitrate (NOx), total oxidant status (TOS), total antioxidant response (TAR), fasting blood glucose, insulin, and homeostasis model assessment of insulin resistance (HOMA-IR.) MHMO have similar hsCRP and TNF-α values as the MUHMO with MS, while chemerin was significantly lower in MHMO. NOx was higher in MUHMO with MS patients, while no difference regarding TOS and TAR was found between the two groups. HOMA-IR and insulin values were lower in MHMO as compared to the MUHMO with MS group. Insulin, HOMA-IR, and chemerin were identified predictors of MS in MHMO. In conclusion, MHMO and MUHMO display similarities and differences in terms of chronic inflammation, nitro-oxidative stress, and IR. Markers of IR and chemerin are possible predictors of MS in MHMO.

## 1. Introduction

Obesity is one of the most important burdens of the health care systems worldwide [1]. It is often associated with type 2 diabetes, cardiovascular diseases, dyslipidemia, metabolic syndrome, a reduced life expectancy, and therefore it is considered a metabolically unhealthy obese (MUHO) phenotype [2,3]. Nevertheless, a distinct subset has been described, i.e., metabolically healthy obesity (MHO), characterised by the presence of obesity in the absence of metabolic disturbances [3,4]. While most studies use as criteria for defining MHO less than 2 conditions of metabolic syndrome (MS), others use a more strict definition, i.e., the absence of type 2 diabetes, dyslipidemia, and hypertension [4,5,6], and some include also markers of homeostasis model assessment of insulin resistance (HOMA-IR) and systemic inflammation markers such as C reactive protein (CRP) [7,8,9]. Hence, at present, as there is no general consensus on the diagnostic criteria, the estimation of its prevalence is quite difficult, studies reporting a wide range between 2% and 50% [2,3,5,10]. 

A large body of work has shown that excess adipose tissue induces adipocyte dysfunction that further causes an imbalance of the production of adipokines such as tumor necrosis factor alpha (TNF-α) or IL-6 involved in the onset of IR and other obesity associated disturbances [11]. Also, a novel adipokine, namely chemerin, has been revealed to be a promotor of inflammation by stimulating macrophage adhesion and therefore a predictor of metabolic syndrome mainly regarding insulin and glucose homeostasis [12]. An obesity low grade inflammatory state has been confirmed by the elevated levels of pro-inflammatory marker CRP a well-known harbinger of type 2 diabetes [13]. Furthermore, obesity is associated with oxidative stress which is linked to chronic inflammation and explains metabolic alterations such as IR and type 2 diabetes [14,15]. 

MHO has been reported to be associated with lower levels of visceral fat, fasting insulin, plasma triglycerides, high-sensitivity CRP (hsCRP), interleukin-6 (IL-6), and tumor necrosis factor alpha (TNF-α) than their MUHO counterparts [16,17]. Thus, MHO seems to be a lower inflammatory state, holding a protective role towards metabolic complications [7]. However, Gomez et al. [18] revealed that circulating concentrations of pro-inflammatory parameters such as CRP are similarly increased in both MHO and MUHO. Therefore, to what extent chronic inflammation accounts for the metabolic differences between MHO and MUHO is still a matter of debate. 

Data from the literature has indicated that MHO are still at risk of cardiovascular diseases [19,20] and type 2 diabetes [21] but the risk is lower as compared to their MUHO counterparts who have worse metabolic profiles [5]. However, Appleton et al. showed that 30.6% of MHO become MUHO over a 10-year period [5,22], pointing out that MHO is a transient state that may change, in time, into MUHO [2,5,23]. Further information regarding cardio-metabolic risk and IR, although not referring to MHO directly, has been provided by the Swedish Obese Subjects (SOS) study that compared conventional treatment with bariatric surgery and showed that the effectiveness of the intervention depended on baseline fasting insulin levels [24]. Therefore, based on this data the question whether the group of MHO is really “healthy” still has to be answered and a stratification based on IR and other metabolic abnormalities might be helpful to reveal potential treatment benefit regarding reduction of cardio-metabolic and mortality risk [5,23]. Hence, the aim of our study was to analyse pro-inflammatory, nitro-oxidative stress, and IR markers in metabolically healthy morbidly obese (MHMO) individuals with respect to unhealthy morbidly obese (MUHMO) and to identify the potential predictors of MS in the MHMO group. 

## 2. Material and Methods

### 2.1. Study Design

A retrospective study was conducted on morbidly obese (MO) patients eligible for bariatric surgery who were examined anthropometrically and biochemically before submission to surgery. Blood samples were collected after overnight fasting and serum was obtained through centrifugation. The tests for hsCRP, TNF-α, chemerin, stable end products of nitric oxide (NO), nitrite and nitrate (NOx), total oxidant status (TOS), total antioxidant response (TAR), fasting blood glucose, insulin, and lipids were run immediately or stored at −80 °C until analysis. 

### 2.2. Study Patients

A total of 72 MO patients were recruited between 2009–2015 form the Second Surgical Clinic from Cluj-Napoca, Romania, selected to be referred to bariatric surgery. They all met the 1991 National Institute of Health (NIH) Consensus Conference Guidelines for bariatric procedures criteria for bariatric surgery i.e., body mass index (BMI > 40 kg/m^2^ or BMI > 35 kg/m^2^ with associated comorbidities. Finally we analysed only 48 patients who fulfilled the criteria for MHMO and MUHMO with MS. The selected patients were divided into two groups MHMO (*n* = 16) and MUHMO with MS (*n* = 32) based on the characteristics that were used to define metabolic health. The remaining 24 MO patients had only one condition out of the 4 that describe MS, so they were excluded from the study. 

The study was ruled under the ethical principles of the Helsinki Declaration and the protocol was approved by the Ethics Committee of the “Iuliu Hațieganu” University of Medicine and Pharmacy from Cluj-Napoca (No. 503/15.12.2011). All the participants were informed about the methods of the study and written informed consent was obtained from all subjects.

### 2.3. Obesity Status and Metabolic Health

Morbid obesity was defined according to the current World Health Organization (WHO) classification as having a BMI ≥ 40 kg/m^2^.

Metabolic health was defined according to 2009 International Diabetes Federation (IDF) [25] and to The third report of the National Cholesterol Education Program Adult Treatment Panel III (NCEP-ATP III) [26] consensus criteria for the MS as follows: (1) high blood pressure, defined as blood pressure ≥130/85 mmHg or drug treatment; (2) high fasting blood glucose level, defined as glucose ≥100 mg/dL or drug treatment for type 2 diabetes; (3) high serum triglycerides, defined as triglycerides ≥150 mg/dL or drug treatment; (4) low high density lipoprotein cholesterol (HDL-C) level, defined as HDL-C < 40 mg/dL in men and <50 mg/dL in women or drug treatment for dyslipidemia. MUHMO with MS were considered when ≥2 of the above criteria were met. In order to define MHMO we excluded waist circumference (WC) because of collinearity with BMI (all the patients had the WC over 102 cm in male and over 88 cm in female respectively) and only those patients who had none of the previous conditions for MS were considered MHMO [4].

### 2.4. Clinical and Laboratory Measurements 

Body mass index (BMI) was calculated as weight in kilograms divided by height in meters squared. 

Fasting blood glucose, total cholesterol (TC), high density lipoprotein-cholesterol (HDL-C), and triglycerides were determined using standard enzymatic colorimetric method on an automatic analyser (Prestige 24i, Tokyo Boeki, Tokyo, Japan).

Fasting serum hsCRP levels were evaluated using an automated system for chemiluminescence method (Immulite 1000, Siemens, Munich, Germany) and a commercially Immulite hsCRP kit (Siemens, Munich, Germany). Using ELISA kits serum TNF-α (Thermo Scientific, Waltham, MA, USA), chemerin (Abcam, Cambridge, UK) and insulin (EMD Millipore, Burlington, MA, USA) levels were measured according to the manufacturer indications using an ELISA plate reader (Organon 230S, Oss, The Netherlands). Coefficients of variation intra- and inter-assay for the tests were below 10%. 

The nitro-oxidative stress was evaluated by serum NOx, TOS, and TAR as previously described [27]. Serum NO concentration was evaluated through its final stabile products, nitrite and nitrate (NOx), using a colorimetric method. The principle of this assay is the reduction of nitrate by vanadium (III), combined with detection by the acidic Griess reaction. Serum TOS was measured using a colorimetric method also. The assay is based on the oxidation of ferrous ion to ferric ion in the presence of various oxidant species in acidic medium and the measurement of the ferric ion by xylenol orange. As for serum TAR, we used a colorimetric method as well, where the hydroxyl radical is produced by Fenton reaction, and the rate of the reactions is monitored by following the absorbance of colored dianisidyl radicals [27].

Homeostasis model assessment of insulin resistance (HOMA-IR) using the following formula HOMA-IR = (fasting insulin µUI/mL × fasting glucose mg/dL)/22.5 × 18 was used to evaluate the IR. Low-density lipoprotein-cholesterol (LDL-C) concentration was calculated by the Friedewald formula.

### 2.5. Statistical Analysis 

Statistical analysis of data was released using R-software environment for statistical computing, v. 3.4.4 (R Foundation for Statistical Computing, Vienna, Austria). 

The observed distributions of qualitative variables were described by absolute frequencies while distributions of quantitative variables were presented using mean ± standard deviation or median (interquartile range: percentile 25%–percentile 75%). 

The characterisation of chronic inflammation (hsCRP, TNF-α, and chemerin), nitro-oxidative stress (NOx, TOS, and TAR) as well as of IR (insulin, HOMA-IR) in MHMO as compared with MUHMO with MS was obtained by using the parametric (Student’s *t* test for independent samples) and non-parametric tests. The non-parametric Spearman correlation coefficient (ρ) was also used to study the degree of correlation between the chronic inflammation, nitro-oxidative stress, and IR markers within the MHMO and the MUHMO with MS groups. 

The influence of chronic inflammation, nitro-oxidative stress, and IR parameters upon the MS occurrence was tested by univariate logistic regression analysis. The effect size of association was quantified by odds-ratio (OR) and 95% confidence interval associated.

Because of missing data, we used the pairwise deletion technique in bivariate analysis. The level of significance α = 0.05 was set for all statistical tests.

## 3. Results

### 3.1. Demographic Data of the Studied Groups

Based on the criteria used to define metabolic health, out of the 72 MO patients, 16 (22.22%) of them were considered MHMO, 24 (33.33%) were considered MUHMO as they fulfilled only 1 criteria of MS, while 32 (44.44%) were MUHMO with MS.

The studied groups were similar regarding age distribution (Student’s *t* test, *t*(46) = 0.95, *p* = 0.349). The mean age was 40.4 ± 9.02 years in the MHMO group and 43.3 ± 10.03 years in the MUHMO with MS group. No significant difference in gender distribution (Chi-square test, *p* = 1.00) was found between the groups. The sex ratio (F/M) was 11/5 in MHMO group versus 22/10 in MUHMO group.

### 3.2. Clinical and Laboratory Measurements

The characteristics of the two groups are presented in Table 1. No significant differences were related to groups BMI values (*p* = 0.155). Our results showed that MHMO and MUHMO with MS have similar hsCRP (*p* = 0.200) and TNF-α (*p* = 0.615) values. However, in MUHMO with MS, chemerin was significantly higher as compared to their MHMO counterparts (*p* = 0.044). There was no difference regarding TOS and TAR between the two groups, but however, a significant difference was observed in terms of NOx levels which were higher in MUHMO with MS patients (*p* < 0.001). As for HOMA-IR (*p* < 0.001) and insulin (*p* = 0.001) we found higher values in MUHMO with MS as compared to the MHMO group. Triglycerides were higher in the MUHMO (*p* = 0.003) and HDL-C in the MHMO (*p* = 0.003) group as expected. Surprisingly, there were no statistically significant differences in terms of TC (*p* = 0.984) and LDL-C (*p* = 0.982) between the two groups. 

In the MHMO group a positive correlation was found between NOx and TAR (*r* = 0.829, *p* = 0.042). Also, positive correlations between NOx and insulin (*r* = 0.453, *p* = 0.045), NOx and HOMA-IR (*r* = 450, *p* = 0.047), while negative correlations chemerin and TAR (*r* = −0.798, *p* < 0.001) were identified in the MUHMO with MS. 

### 3.3. Predictors of MS in the MHMO

The results of the regression analysis showed that fasting insulin concentration and HOMA-IR were significant predictors of the MS in MO patients and therefore they are useful tools in differentiating MHMO from MUHMO with MS (Table 2). Also, we found that the elevated level of chemerin is a risk factor for MS in MHMO patients (*p* = 0.023). 

## 4. Discussion

A special subset of obese without metabolic disorders named MHO has been described, but, due to the inconsistency in defining this category, clearer criteria are needed to determine whether an individual is really metabolically healthy [5,8,28]. Given this question, and with the purpose to better characterize MO patients in order to shed light on the further metabolic benefits of bariatric surgery, the present study aimed to analyse and compare in terms of pro-inflammatory, nitro-oxidative and insulin-resistance markers two groups of MHMO and MUHMO with MS and to identify the potential predictors of MS in the MHMO group. Our findings showed that: (1) MHMO patients have similar pro-inflammatory and nitro-oxidative stress profile as the MUHMO with MS patients in terms of hsCRP, TNF-α, TOS, and TAR, while chemerin and NOx are significantly different (lower in MHMO). Also, HOMA-IR and insulin are significantly lower in MHMO. (2). Fasting insulin, HOMA-IR, and chemerin were identified as possible predictors for SM in the MHMO. 

In our study, we identified 22.22% MHMO, a percentage similar with some of the data provided by other authors [29,30]. Goday et al. [29] classified as MHMO if only one or no cardio-metabolic factors were present: high blood pressure, triglycerides, blood glucose (or use of medication for any of these conditions), decreased HDL-C levels, and HOMA-IR >3.29 and reported 18.9% patients who fulfilled the criteria. Moreover, excluding MO patients with diabetes, hypertension, and elevated fasting triglycerides, Lee CJ et al. [31] reported a percentage of 16.9% MHMO, while Haskins et al. [32] considered MHMO only 7% i.e., those who did not meet any criteria for hypertension, dyslipidemia, and insulin resistance defined as a fasting blood glucose level ≥100 mg/dL, glycosylated haemoglobin ≥5.7%, or the use of antidiabetic medications. Finally, Pelascini E et al. [30] reported 20.6% MHMO using more strict criteria according to Wildman et al. excluding MO patients with more than 2 conditions of MS, HOMA-IR over 2.5, and systemic inflammation by CRP above 5 mg/dL. Because of the inconsistency regarding the criteria for the definition of MHO, results from the literature are various and therefore a general consensus on this matter is warranted. In the present study we considered MHMO only those patients displaying none of the criteria of MS following the proposal of a harmonizing definition by Ortega el al [4]. 

A high amount of visceral adipose tissue is a source of the pro-inflammatory cytokines and systemic nitro-oxidative stress through inducible/inflammatory nitric oxide synthase (iNOS) activation, altered mitochondrial function, and endoplasmic reticulum (ER) stress in adipocytes, which both interrelate and makes it difficult to precisely trace which one precedes the other [14,33]. The overproduction of superoxide ions in obese may react with NO and form reactive nitrogen species (RNS), such as peroxynitrite resulting in cellular and organ damage [14]. Therefore, NOx, the indirect marker of NO production, can be used both as a tool of nitro-oxidative stress and chronic inflammation. Moreover, chronic inflammation and oxidative stress seem to be important players of the link between excess adipose tissue and the development of type 2 diabetes (T2DM), hypertension, dyslipidemia, and cardiovascular disease, altogether defining the MS whose cornerstone is IR [7,14,33,34]. However, it is not clear if all the obese subjects carry a similar elevated inflammatory state and data form the literature is conflicting. In the present study we found no significant differences of the hsCRP and TNF-α values between MHMO and MUHMO with MS. Our results are in agreement with other authors who also studied MO patients [18,30]. Furthermore, Molli et al. showed the presence of a similar degree of inflammation, measured by hsCRP levels, when comparing two large populations of MHO with obese with MS [35]. However, lower CRP, TNF-α, and IL-6 levels have been detected in MHO individuals relative to their MUHO counterparts by other authors [16,17,36]. Wildman et al. [37] showed that overweight/obese women without clustering of cardio-metabolic risk factors and diabetes have elevated inflammatory biomarkers, but, those with both excess body size and cardio-metabolic risk factors have the greatest burden of inflammation. Also, the study shows that both excess adipose tissue and cardio-metabolic abnormalities are independently associated with the inflammatory profile [37].

Chemerin, an adipocytokine with a chemoattractant role that is involved in glucose and lipid homeostasis was demonstrated to be elevated in obese individuals and to promote inflammation [38,39,40,41]. We showed that chemerin level was significantly lower in MHMO as compared with MUHMO with MS, opposite to Alfadda who found no differences between the two groups [42]. Chemerin has been proved to be associated with other pro-inflammatory markers such CRP, and TNF-α and has been suggested to be a predictor of MS, particularly regarding insulin and glucose homeostasis [43,44]. Correlations between circulating chemerin and IR have been demonstrated also by Catalan et al. [12]. Herein we showed that higher levels of chemerin are a significant predictor and more precisely a risk factor for the onset of MS in MHMO. Also, we observed a negative correlation between chemerin and TAR in MUHMO suggesting that an increased chronic inflammation and IR is accompanied by a reduction in the antioxidant defence. This data is significant as in MO patients submitted to bariatric surgery reduction of chemerin serum concentrations was associated with weight loss and improvement of metabolic markers [12]. 

One possible explication for the differences between the chronic inflammation level between MHMO and MUHMO with MS may reside from the amount of abdominal adipose tissue. In this respect, some authors have shown that at-risk obese individuals have more abdominal adiposity than MHO despite similar BMI values [9,45]. Furthermore, visceral adipose tissue is known to be associated with higher content of macrophages in insulin resistant individuals as compared to insulin sensitive ones, being responsible for a higher chronic inflammation [46]. However, van Beek et al. [47] argued that the increased inflammatory state (they found no differences in CRP and TNF-α, but higher IL-6 levels in MO with type 2 diabetes as compared with MHMO) could be explained by differences in adipose tissue mass or fat distribution, as they observed that the adipocyte sizes were not significantly different between the two groups. Secondly, it may be that some pro-inflammatory markers are elevated in MO with comorbidities (see chemerin) while others are not (CRP and TNF-α). Thirdly, the association between chronic inflammation and metabolically healthy obesity is definition dependent [48]. 

The damage induced by oxidative stress occurs only when the antioxidant systems are unable to neutralize the overproduction of ROS [49,50]. Interestingly, herein, we observed that MHMO patients have a similar oxidative stress profile in terms of TAR and TOS as the MUHMO with MS patients. However, the level of NOx induced by iNOS upon stimulation by inflammatory cytokines was significantly lower in MHMO as compared to MUHMO with MS. We have previously showed that TOS, TAR, and NOx are elevated in MO patients as compared to normal weight healthy individuals [27]. Elevated levels of NOx have been found in severely obese children, with higher circulating levels in those with more than four metabolic risk factors [51]. Opposite to our results, Bañuls C et al. showed that total and mitochondrial ROS (reactive oxygen species) production were significantly higher in the metabolically abnormal MO and metabolically abnormal MO with type 2 diabetes groups than in the MHMO group but, total antioxidant capacity showed non-significant difference confirming our findings [52]. Furthermore, Lwow F et al. found that both antioxidant activity and oxidative stress (evaluated by TBARS) in postmenopausal obese women depend on obesity phenotype as antioxidant activity in MUHO individuals was significantly lower and thiobarbituric acid reactive substances (TBARS) concentration was significantly higher when compared with their MHO counterparts [53]. Moreover, oxidative stress is known to be related to IR and diabetes [14]. Although we found a significant difference in the IR degree between the two groups, it did not, however, run in parallel with a significant difference of TAR and TOS between MHMO and MUHMO. Hand in hand with our results, Tinahones FJ et al. reported that the concentration of lipid peroxidation evaluated by TBARS as well as fasting plasma antioxidant capacity was similar in two groups of severely obese patients independently of the state of IR [50]. Finally, a possible explanation for the lack of evidence of differences in oxidative stress between the two groups of patients could be the absence of identification of the possible elevation of antioxidant enzymes such as superoxide dismutase, glutathione peroxidase, and catalase as they were not determined in our study. TAR encompasses only non-enzymatic antioxidants, respectively endogenous molecules (e.g., uric acid, albumin) and exogenous antioxidants derived from the diet (e.g., ascorbic acid, vitamin E) [50]. 

Finally, we observed that fasting insulin and HOMA-IR were significantly lower in MHMO as compared to MUHMO with MS which is in line with other reports [35,42]. One possible explanation of the difference might reside from the results of Brochu et al. [54] who showed that women with MHO had 50% less visceral adipose tissue than the MUHO postmenopausal obese women and displayed greater insulin sensitivity suggesting that a smaller amount of visceral adipose tissue is a significant factor in the maintenance of the favourable metabolic profile of MHO [17]. Genetic studies have shown that the adipose tissue storage and expandability capacity may be the milestone in the development of metabolic abnormalities [55]. It may be that MHO holds a greater flexibility and ability to manage the dietary overload as compared to the MUHO individuals [17]. 

Although there are some differences between MHO and MUHO regarding fat distribution patterns (less ectopic and visceral fat in MHO), angiogenesis potential, and macrophages infiltration/activation [5,37,56], it has been demonstrated that MHO is not a stable state and that, within time, it can shift towards metabolically unhealthy obesity [2,5,23]. In fact, in our study, we demonstrated that fasting insulin, HOMA-IR, and chemerin are univariate predictors for MS in MHMO. More precisely, Mongraw-Chaffin et al. showed that both duration and severity of obesity are positively associated with incident MS, underlining that MHO is a transient state in the pathway to cardio-metabolic disease [23]. Therefore, due to this dynamic feature of MHO, we may argue that MHMO patients would benefit from bariatric surgery as its aim is to obtain and sustain weight loss and hence to preclude the onset of metabolic disturbances. 

The present study has some limitations. First, our work included a low number of patients. Second, we did not include the WC or waist to hip ratio data in our analysis, so we were not able to draw conclusions on the associations between chronic inflammation, nitro-oxidative stress, and IR markers and abdominal obesity. Third, we have to keep in mind that due to the inconsistency in the definition criteria of MHO the results must be interpreted as such. Finally, the cross sectional study permits the estimation of some indicators such as OR (therefore the prediction) but the interpretation must be cautious and future longitudinal studies must endorse the validation of the tested model. Also, the validation of these factors as independent predictors of MS in the context of the presence of other covariates deserves further investigation for their predictive value of MS apparition in MHMO in appropriately designed intervention studies. 

## 5. Conclusions 

In conclusion, our study showed that MHMO and MUHMO display similarities and differences in terms of pro-inflammatory and nitro-oxidative stress profile while MHMO are less insulin resistant than MUHMO. Furthermore, fasting insulin, HOMA-IR, and chemerin might be predictors of MS in MHMO, but this finding has to be confirmed in larger longitudinal studies and adjusted for potential confounders in further studies. 

## Figures and Tables

**Table 1:** Characteristics of the studied groups.

	MHMO	MUHMO	*p*-Value *
BMI (kg/m^2^) ^a^	45.61 ± 7.67	49.04 ± 7.80	0.155
hsCRP (mg/L) ^b^	12.05 (4.62–15.77)	13.36 (7.52–17.64)	0.200
TNF-α (pg/mL) ^b^	35.45 (30.21–40.35)	34.22 (24.4–47.08)	0.615
Chemerin (ng/mL) ^b^	30.74 (23–43.71)	52.5 (34.89–92.2)	0.044
NOx (μmol/L) ^a^	54.86 ± 7.14	71.77 ± 9.03	<0.001
TOS (μmol H_2_O_2_ equiv./L) ^a^	86.03 ± 31.20	92.60 ± 25.44	0.605
TAR (mmol trolox equiv./L ^b^	0.61 (0.6–0.62)	0.61 (0.49–1.09)	0.882
Fasting glucose (mg/dL) ^b^	85 (79.50–89)	109 (101–123.5)	<0.001
Fasting insulin (μUI/mL) ^b^	6.22 (2.12–9.59)	12.85 (8.57–18.92)	0.001
HOMA-IR ^b^	1.37 (0.67–1.90)	3.56 (2.55–5.65)	<0.001
TC (mg/dL) ^a^	203.3 ± 29.46	203.03 ± 52.68	0.984
Triglycerides (mg/dL) ^b^	102.5 (83–125)	162.5 (119–205)	0.003
HDL-C (mg/dL) ^b^	62.4 (53.5–70.3)	40 (37.1–49.20)	0.003
LDL-C (mg/dL) ^a^	124.55 ± 40.6	125.04 ± 53.56	0.982

MHMO: metabolically healthy morbidly obese, MUHMO: metabolically unhealthy morbidly obese, BMI: body mass index, hsCRP: high sensitivity C-reactive protein, TNF-α: tumor necrosis factor alpha, NOx: nitrites/nitrates, TOS: total oxidant status, TAR: total antioxidant response, HOMA-IR: homeostasis model assessment of insulin resistance, TC: total cholesterol, HDL-C: high density lipoprotein cholesterol, LDL-C: low-density lipoprotein cholesterol; ^a^ mean ± standard deviation; ^b^ median (interquartile range: percentile 25%–percentile 75%); * Student for independent groups or Mann–Whitney’s test.

**Table 2:** Results of univariate binomial logistic regression analysis.

	COR	95% CI	*p*-Value
hsCRP (per 1 mg/L increase)	1.03	0.97–1.08	0.352
TNF-α (per 1 pg/mL increase)	1.01	0.98–1.03	0.712
Chemerin (per 1 ng/mL increase)	1.02	1.00–1.04	0.058
Chemerin (qualitative)			
Tertile 1	Reference	Reference	-
Tertile 2	1.91	0.45–7.98	0.378
Tertile 3	**8.00**	**1.33**–**48.18**	**0.023**
Fasting insulin (per 1 μUI/mL increase)	**1.18**	**1.04**–**1.34**	**0.011**
HOMA-IR (per 1 point increase)	**2.75**	**1.42**–**5.30**	**0.003**
NOx (per 1 μmol/L increase)	1.72	0.98–3.01	0.058
TOS (per 1 μmol H2O2 equiv./L increase)	1.01	0.98–1.05	0.589
TAR (per 1 mmol trolox equiv./L increase)	1.36	0.03–54.94	0.869

COR = crude odds-ratio; CI= confidence interval; bold values denoted statistical significance; tertile 1: (13.71; 32.04) ng/mL; tertile 2: (32.04; 60.16) ng/mL; tertile 3: (60.16;217.25) ng/mL.
